# Knowledge, Attitudes, and Perceptions of Occupational Health and Safety Among Migrant Agricultural Workers in Ontario, Canada: A Pilot Study

**DOI:** 10.3390/ijerph23070906

**Published:** 2026-07-15

**Authors:** Craig Fairclough, Janet McLaughlin, Jaskaren Randhawa, Chun-Yip Hon, Abigail K. Leonard

**Affiliations:** 1 Workplace Safety & Prevention Services, 5110 Creekbank Road, Mississauga, ON L4W 0A1, Canada; 2 School of Occupational and Public Health, Toronto Metropolitan University, 350 Victoria Street, Toronto, ON M5B 2K3, Canada; 3 Department of Health Studies, Wilfrid Laurier University, 73 George Street, Brantford, ON N3T 2Y3, Canada

**Keywords:** migrant worker, agriculture worker, occupational health and safety

## Abstract

**Highlights:**

**Public health relevance—How does this work relate to a public health issue?**

- This work relates to public health by highlighting continuing concerns regarding living and working conditions faced by migrant agricultural workers (MAWs) when they come to Canada for employment.
- Several participants reported awareness of positive COVID-19 cases; however, their attitudes toward this risk warrant further attention to inform planning for future outbreaks.

**Public health significance—Why is this work of significance to public health?**

- This work sheds light on the need to further protect MAWs due to the occupational health and safety (OHS) hazards to which they are exposed.
- In many cases, language and cultural barriers hinder effective communication of OHS controls.

**Public health implications—What are the key implications or messages for practitioners, policy makers and/or researchers in public health?**

- Policy makers and public health researchers can use the findings from this study to strengthen health and safety strategies aimed at better protecting MAWs.
- Similarly, policy makers can use these findings to address workers’ vulnerability stemming from limited awareness of their basic OHS rights.

**Abstract:**

Migrant agricultural workers (MAWs), predominantly individuals from countries in the Global South, play a vital role in maintaining Canadian food security. Employed in low-income positions across the country, they often face workplace hazards and numerous occupational health and safety (OHS) challenges related to long working hours, limited access to OHS information, gaps in knowledge, structural power imbalances with employers, and language and cultural barriers. This pilot study explored the knowledge, attitudes, and perceptions of OHS issues among MAWs in Southern and Eastern Ontario during the COVID-19 pandemic. Participants completed survey questions on working conditions, OHS hazards, and living conditions. A total of 93 questionnaires were completed, 55 by Spanish-speaking and 38 by English-speaking individuals, with 91% of respondents identifying as male. Several participants reported awareness of positive COVID-19 cases in their workplaces, and some indicated having experienced OHS-related illnesses or injuries but felt uncomfortable reporting them to supervisors. Knowledge of OHS rights varied, with a notable minority uncertain about their entitlement to sick leave. The findings indicate a need for further research and targeted interventions to strengthen health and safety practices among MAWs and to ensure they are informed, protected, and supported in exercising their workplace rights.

## 1. Introduction

In 2023, Canada employed nearly 950,000 temporary foreign workers, with close to 80,000 working in agriculture [1,2]. Most of these agriculture workers came from countries in the Global South, notably Mexico, Guatemala, Jamaica, India, and the Philippines [1]. Among the various programs facilitating this labor migration, the Seasonal Agricultural Worker Program (SAWP) is the longest standing, in place since 1966, and has seen consistent growth over the years, bringing in approximately 36,000 workers in 2023 [3]. The SAWP is based on bi-lateral agreements with Mexico and several Commonwealth Caribbean countries and employs workers on contracts of up to eight months annually. In contrast to the SAWP, the general agricultural stream of the Temporary Foreign Worker Program (TFWP) may employ workers from any country for up to two years at a time.

Migrant agricultural workers (MAWs) are employed on temporary contracts tied to specific employers on whose property they typically live, with no job security and no direct pathway to permanent residency. These workers experience health and safety hazards arising from substandard living and dangerous working conditions in Canada [4,5,6]. In addition, MAWs face myriad barriers to accessing health care, such as language and cultural differences, social and physical isolation, and fear of loss of employment. In 2020, 12 percent of MAWs tested positive for COVID-19 and three died in Ontario alone [7], while nine MAWs died in Ontario between January 2020 and June 2021 [8]. The experiences of MAWs have garnered much attention over the last two decades, but relatively little has been researched about the occupational health and safety (OHS) experiences of these workers in Southern and Eastern Ontario Canada since the pandemic began.

According to Ujita et al. [9], MAWs are commonly employed in what are known as the “3D” job sectors—dirty, difficult, and degrading. These roles are typically seasonal and carry elevated risks to occupational health and safety [9]. This framework could be expanded by adding dangerous and demanding, further highlighting the harsh conditions often faced by workers in these environments. MAWs in Canada experience harsh working conditions because of the industry in which they are employed as well as the conditions of their contract. When compared with other industries provincially and nationally, agriculture is acknowledged as a dangerous occupation, ranking among the most hazardous in terms of fatalities [10]. Hazards such as chemical and climatic exposures, in addition to working with machines and in awkward and physically demanding positions for long periods of time without sufficient rest or breaks, generate vulnerability to injury and illness. With respect to their contract, MAWs are bound to specific employers; their ability to stay and work in Canada thus depends on maintaining a positive relationship with them, creating a uniquely unbalanced power relationship [11].

Migrant workers are among the most precariously positioned in the economy, with research indicating that many are reluctant to take time off when ill due to fear of job loss [12]. One underlying motivation for this behavior is the economic necessity driving their migration: most of these workers leave their home countries to work in more affluent nations in order to send remittances to their families due to a lack of employment prospects in their countries of origin [13]. This financial responsibility often compels them to continue working despite health concerns, sometimes at great personal cost [14].

A systematic review by Hargreaves et al. [15] reveals numerous barriers international migrant workers experience in accessing health care, as well as social isolation and mental health concerns. In Canada, research has consistently revealed similar concerns. In Ontario, agricultural workers are excluded from several components of the Employment Standards Act (including provisions such as hours of work, rest periods, and overtime) as well as the Labour Relations Act [4]. Although legally employed migrant workers in Ontario have access to provincial health care and workers’ compensation, they experience myriad structural and practical barriers that often render such access unattainable [16,17]. In the midst of long hours of work, transportation and language barriers, they often need to rely on employer facilitation to access care; however, due to structurally imbalanced and precarious employment relationships, many workers do not feel comfortable asking for time off [18]. Earlier research revealed that migrant workers also face numerous OHS risks, but their attitudes about such risks have not been thoroughly assessed in relation to the COVID-19 pandemic [4]. The objective of this study is to draw attention to the level of knowledge (what they know), attitudes (how they feel), and perceptions (a viewpoint) of the OHS issues among MAWs working in the Southern and Eastern Ontario Canada region amid the pandemic [17]. This data will be beneficial to plan and mitigate prior to another outbreak occurring in the future.

## 2. Materials and Methods

This was a survey-based cross-sectional study in which institutional ethics approval was granted prior to any data collection (Research Ethics Board #2022–2025). The survey was arranged into three sections. The first section contained demographic questions such as age range, sex, level of education, and country of origin as well as questions about the migrant worker and their work, including whether they have ever been injured at work. The second section addressed questions related to their thoughts about safety in their workplace and how their supervisor handles health and safety issues such as reporting health and safety issues expectations and provision of health and safety information. The third section addressed living conditions while working as a migrant agriculture worker such as having access to drinking water and suitable housing conditions. In the second and third sections of the questionnaire, the respondents noted their level of agreement with the questions on a five-point Likert scale ranging from strongly agree (1) to agree (2), disagree (3), strongly disagree (4), and don’t know (5). The five-point Likert scale was used to ensure ease and efficiency for the workers completing the questionnaire.

The questions were first shared with farming consultant experts to ensure that they were relevant and unambiguous to a specific employee, as well as length-appropriate. The questions were translated into Spanish using Google Translate (Mountain View, CA, USA) and then verified by two Spanish-speaking interpreters. This ensured that the appropriate version could be provided to MAWs, the majority of whom speak either English or Spanish. The inclusion criteria were for any English or Spanish-speaking MAW in Southern or Eastern Ontario Canada. The questionnaire was administered at farming workplaces and on site at community events between 12 August 2022 and 16 July 2023. Participants were sampled via convenience sampling. Various community outreach events, such as cricket and soccer matches that MAWs attend, as well as three farming workplaces, were sought out where participants were recruited to complete the survey. Informed consent was requested and granted before the participants were handed the questionnaire for completion. Most workers completed the survey independently. Some relied on their colleagues, and some asked for clarification from the administrators of the survey. Most completed the questions within five minutes. The questions were developed for this study and partly adopted from a previous body of work done by Otero and Preibisch [6]. No validity nor reliability testing was performed in this study.

The questionnaire consisted of structured, predetermined questions presented in the same sequence for all participants. The responses were closed-ended (e.g., yes/no, multiple choice). This was to maximize clarity, reliability, and comparability across respondents. The data analysis used descriptive statistics and cross-tabulations. The data analysis was performed using Microsoft Excel (Redmond, WA, USA). Data storage was declared to the Research Ethics Board as per their guidelines.

## 3. Results

### 3.1. Demographics

In total, there were 93 questionnaires completed, 55 in Spanish and 38 in English. A total of 92 of 93 respondents provided a response to respective age range (Table 1). There were 83 of 91 respondents to the gender question; 91% identified as male, 8 of 91 (9%) female, and 2 respondents did not identify a gender. Of the 93 questionnaires completed, 83 respondents completed the self-identification question: 37 (45%) self-identified as Black, 18 (22%) self-identified as a person of color, 2 (2%) self-identified as white, and 26 (31%) self-identified as other, while 10 individuals did not answer this question.

For the country of origin, 54 of 93 respondents (58%) were from Mexico, 1 of 93 (1%) from Central America, and 38 of 93 (41%) from the Caribbean. With respect to level of education, 89 completed the question, of which 44 (49%) completed secondary school, 13 (14.6%) completed post-secondary, and 32 (36%) did not finish secondary nor post-secondary education. Participants were asked on a scale from 1 to 10 (1 is do not like and 10 is like very much) how much they like being a farm worker. A total of 17 out of 92 (18%) respondents indicated 7 or lower, while 75 out of 92 (81%) indicated 8 or higher.

### 3.2. Health and Safety-Related Question Results

Survey responses regarding occupational health hazards—such as excessive noise and heat stress encountered during farm work—revealed that 59 of the 83 participants who provided a response (71%) reported no such experiences (Table 2). In contrast, 13 of 83 respondents (16%) indicated experiencing health-related hazards between 1 and 5 times, 10 of 83 respondents (12%) reported occurrences ranging from 6 to 10 times, and 1 of 83 respondents (1%) noted experiencing such hazards more than 10 times.

Regarding safety hazards, including incidents such as falls or cuts sustained while working on the farm, 67 of 92 participants (73%) reported no experience of safety-related incidents. Meanwhile, 22 of 92 respondents (24%) indicated experiencing safety hazards between 1 and 5 times, and 3 of 92 respondents (3%) reported experiencing them between 6 and 10 times.

**Figure 1:**
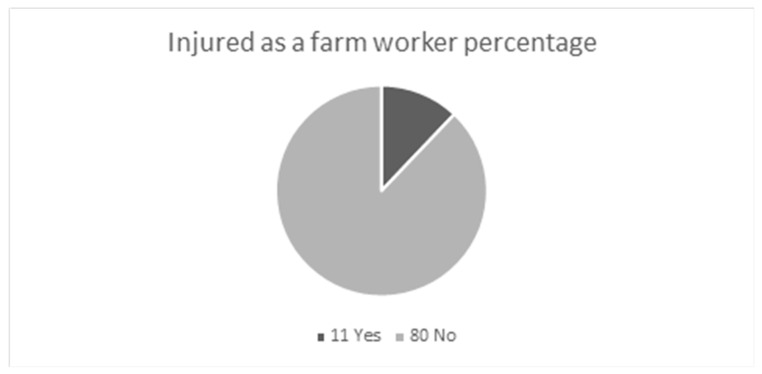
The number and percentage of workers reporting an injury.

A total of 11 out of 91 respondents (12%) (Figure 1) reported having sustained an injury while working on the farm, whereas 80 out of 91 respondents (88%) indicated they had not experienced any farm-related injuries.

Participants were asked if they were aware of any confirmed COVID-19 cases on the farm where they work within the last year and 91 provided a response; 68 (75%) of the respondents indicated no and 23 (25%) indicated that they were aware of COVID-19 cases present at their workplace.

### 3.3. Living and Working Conditions as a Migrant Agriculture Worker

Responses to questions concerning workplace safety and benefits revealed mixed levels of awareness and access among agricultural workers. Of the 92 respondents who addressed the question regarding chemical safety, just over half (52%, *n* = 48) (Table 3.) agreed that they knew how to work safely with the chemicals sprayed on crops. However, a significant portion (40%, *n* = 37) reported uncertainty, and 7.6% (*n* = 7) disagreed. Regarding paid sick leave, 74% (*n* = 67) of the 91 respondents indicated they could take a paid sick day if ill, while 8% (*n* = 7) disagreed and 19% (*n* = 17) were unsure. When asked whether supervisors seek employee input before making health and safety decisions, 86% (*n* = 78) of the 91 respondents agreed, whereas 11% (*n* = 10) disagreed. These findings suggest that while many workers feel included in safety-related decision-making, gaps remain in knowledge and clarity around chemical safety and entitlements such as paid sick leave.

Survey responses indicate generally positive perceptions of workplace conditions among agricultural workers. Of the 91 respondents who answered the question regarding access to drinking water, 92% (*n* = 84) agreed that they had easy access, while 8% (*n* = 7) disagreed. Similarly, among the 89 respondents who addressed the provision of protective measures against environmental exposure, 85% (*n* = 76) agreed that they were given protection to work in the rain and sun, with 12% (*n* = 11) expressing disagreement. When asked about job security in the context of reporting workplace injuries, 89% (*n* = 82) of the 92 respondents indicated they were not afraid of losing their job if they informed their supervisor about an injury, whereas 9% (*n* = 8) disagreed.

## 4. Discussion

This study aimed to examine the occupational health and safety (OHS) experiences of migrant agricultural workers (MAWs) in the Southern and Eastern regions of Ontario, Canada. It also sought to explore these workers’ knowledge, attitudes, and perceptions regarding OHS-related issues. The findings indicate a variable understanding of such issues among these workers. Although the majority of MAWs appeared to be aware of the basic health and safety rights and benefits covered in this questionnaire, a significant minority indicated that they did not feel well protected or knowledgeable. Previous research has uncovered higher levels of concern [4,5,16,19], suggesting that some improvements may have been made in recent years, potentially related to the heightened health and safety protocols emerging in relation to the pandemic. Nonetheless, the limited understanding and awareness of OHS issues observed among some participants may be attributed to an ongoing lack of prior exposure to workplace safety education and protocols, a finding echoed from earlier studies.

During the coronavirus pandemic, certain sectors of the workforce were designated as essential, including health care, food and beverage services, manufacturing and distribution, and agriculture. These sectors relied heavily on migrant labor, such as the workers surveyed in this study [20]. COVID-19 outbreaks were notably prevalent within these industries across several countries, including Italy, France, Spain, and the United States [8]. In Canada, Weikle [21] reported significant concerns in Southern Ontario during the first wave of the pandemic, resulting in over 500 confirmed cases and three fatalities among agricultural workers [8,16,21]. Indeed, MAWs were identified as one of the most vulnerable groups to COVID-19 in Canada [7,22]. It is therefore unsurprising that 25% of respondents in this study (conducted in 2022–23) indicated awareness of COVID-19 cases on their farms within the past year, and it is likely that many more cases went undetected. These findings underscore the continued relevance of COVID-19, particularly in light of emerging variants, and highlight the importance of maintaining public health protocols within essential sectors.

Participants in this study were asked on a scale from 1 to 10 (1 is do not like and 10 is like very much) how much they liked being a farm worker. Less than 20% indicated 7 or lower, while 75 (81%) indicated 8 or higher. These results must be read with caution. First, the question itself may have been ambiguous for some respondents—workers might like certain aspects of employment (e.g., the chance to earn substantial remittances for their families), while disliking other aspects (e.g., family separation). Moreover, many MAWs fear saying anything negative about their experiences due to concern over losing current or future employment, due to the extreme precarity of their contract, in which both their job and ability to stay in Canada are mediated by a single employer [23]. Obokata (2023) noted similar sentiments where workers feared reporting abuse because they were afraid that such action could lead to job loss and deportation [24].

Several respondents reported experiencing health-related and/or safety hazards at least once during their employment. This finding is particularly concerning given that similar issues were documented nearly a decade earlier, highlighting comparable risks [4,17,23]. The persistence of these concerns over time underscores the need for continued investigation into their underlying causes. Further research is essential to identify systemic factors contributing to these hazards and to develop comprehensive strategies for their mitigation.

One pervasive risk in agricultural work is climatic exposures, such as sun, cold, and rain [12]. Farming is among the work sectors that experience high levels of ultraviolet radiation due to sun exposure [1]. Over 12% of the respondents indicated they were not provided with protection from the sun. Outside of year-round operations such as greenhouses, the vast majority of agricultural work takes place during the non-winter months [25]. Adverse health effects associated with sun exposure on those working outdoors include heat stroke and skin cancer [26].

Approximately 10 percent of respondents indicated fear of job loss if they reported a workplace injury to their supervisor. This finding is concerning, as it challenges the expectation that all workers should feel safe and supported in disclosing health and safety concerns. Substantial literature suggests that MAWs experience heightened vulnerability due to their precarious employment status and the financial dependence of family members who depend on Canadian employment [27,28]. Such circumstances can create a compounding effect, where workers continue to labor through early-stage injuries, potentially exacerbating their condition and leading to long-term disability [17,29]. Ensuring that workers feel empowered to report OHS issues is critical. This right is enshrined in the Ontario Occupational Health and Safety Act [30], and its enforcement is essential to safeguarding worker well-being [4].

Some participants in this study may have been concerned about potential repercussions from their employer and therefore were guarded in their responses. Also, because of the recruitment methodology, the participants who chose to complete the questionnaire may not be a full representation of migrant agricultural workers across all of Ontario. In particular, those attending sporting and community events may have been more socially connected than other MAWs, given that the most isolated workers may not have attended such events. This introduces the possibility of response bias towards potentially more positive experiences and is a possible limitation of the study. The limited sample size, limited generalizability, and no formal questionnaire validation are further possible limitations of the study.

Further, this study showed that more than a quarter of the migrant workers surveyed do not know if they can take time off work when they fall ill. Possible reasons for this finding include employers failing to make this right clear, or workers may fear retribution from employers if they request time off. This is particularly concerning, since working through injuries or illness can compound and exacerbate problems, leading to more serious concerns [31]. Similarly, in an earlier study, Hennebry et al. [16] found that multiple barriers impede health care access. Approximately half of the nearly 600 workers surveyed said they would not seek medical care when sick in order to not lose work hours; 44% indicated their co-workers would not take time off work for health care due to fear of asking their employer for permission; and 27% feared loss of employment over a health problem [16].

In this study, 29% of participants indicated that they experienced health hazards and 27% noted that they have experienced safety hazards while working. These findings support other research demonstrating that MAWs are exposed to numerous hazards leading to injuries and or illnesses, which can be both acute or chronic [16,17,32]. Agriculture is among the Canada’s most dangerous industries, but MAWs face compounded vulnerabilities due to their precarious employment status, in which they may not feel comfortable or empowered to raise concerns, and a lack of training regarding health, safety, and OHS protections and rights, which may be compounded by language barriers [14]. One study noted that workers have a fatalistic attitude towards workplace accidents, suggesting that workplace injuries and accidents are inevitable [33]. This may be a similar attitude with this cohort of workers as well.

An examination of migrant workers across sectors beyond agriculture reveals a consistent pattern of OHS challenges and vulnerabilities. Cedillo et al. [34] found that migrant workers in the hospitality, fast food, meat processing, and construction industries reported OHS concerns. Likewise, Porru et al. [31] identified a persistent challenge faced by migrant workers in reconciling the pursuit of gainful employment with the maintenance of their health, irrespective of industry. Two key contributing factors include limited access to health-related information and a tendency among migrant workers to underestimate occupational risks and engage in higher-risk behaviors [17]. These findings led Porru et al. to propose that migrant status itself should be recognized as a distinct social determinant of health [31].

While the scope of this study was limited to migrant workers in Ontario agriculture, work conducted in other provinces uncovered similar concerns. For example, in British Columbia, Otero and Preibisch [6] found language barriers, lack of information, and knowledge of rights, among other factors, that “are intimately linked to people’s ethnicity and citizenship” [6] (p. 4). Likewise, in a comparative study of workers in Ontario and Quebec, MAWs were found not only to face myriad OHS risks, but also numerous barriers to return to work upon injury [29].

In Alberta, a study conducted by Shankar et al. [14] highlights immigrant workers’ lack of understanding of OHS. While workers indicated they were familiar with their right to refuse unsafe work, they were afraid to exercise their right to refuse work for fear of reprisal up to and including termination. Additionally, workers were not familiar with their other two rights, which include the right to know and the right to participate [14].

Although most of the workers in this current study indicated they were clear about their rights and responsibilities in relation to workplace health and safety, a considerable number indicated they were not. This study, then, adds to a growing body of literature suggesting that immigrant and migrant workers, across geographic contexts as well as industries, are profoundly vulnerable to OHS concerns with inadequate rights protections.

While this study’s scope was limited to Ontario, Canada, it has relevance to migrant workers internationally. In various geographic contexts, migrant workers from countries of the Global South are integrated into agricultural production in the Global North. For example, the American Public Health Association [35] highlights that more than 50% of the dairy workers in the United States are refugees and immigrants. In New York and Wisconsin, more than 75% of dairy workers are Spanish-speaking, primarily from Guatemala and Mexico. Refugees from countries in Africa such as Somalia and Egypt work in meat and poultry processing [35].

## 5. Practical Application

The findings from this study can carefully inform appropriate policies, programs, and practice improvements directed at enhanced health and safety strategies for MAWs while considering the sampling strategy and limited generalizability. Benefits can be gained and health and safety conditions improved by empowering such workers and reducing their sense of vulnerability. This includes employers facilitating health and safety training, engaging workers in open conversation, and consulting with representatives to determine strategies to decrease vulnerability. These opportunities will offer supervisors key insights that can act as discussion points and scenarios that will help contextualize the lessons to meet the unique needs of migrant workers.

Recognizing the unique cultural barriers facing migrant workers and the lack of adequate information, education, and training, cultivating a culture of safety among this population will require an awareness of all hazards and the mandatory controls in place to address them. It is critically important that employers develop training programs, both during onboarding and as refresher sessions, to ensure workers have a solid understanding of health and safety hazards and appropriate controls. Of particular concern is awareness of and adherence to recommended safety precautions regarding chemicals used for crop protection, engine maintenance, veterinary care, and other applications in agricultural settings, with more than 47% of survey respondents indicating they are unaware of appropriate procedures for working with chemicals. Chemical safety training and adequate personal protective equipment (PPE) should be provided to all workers who are exposed to chemicals through handling, application, storage, disposal and potential remediation. Beyond a single educational session, training should be revisited periodically with regular knowledge checks to ensure thorough understanding of appropriate protocols. The recommended approach for migrant workers in the agricultural sector would be applicable for all industry sectors where chemical exposure is experienced. To ensure a comprehensive understanding of safety precautions, employers should consider delivering the training in different modes to satisfy the different learning styles as well as accommodate language proficiency. Multilingual resources, including translated training materials for Spanish-speaking workers, will increase comprehension of training content. Relaying vital information through pictograms can also support workers who may have trouble comprehending the verbiage in the training delivery.

More than 10% of workers in this study noted that they were not provided with any sun safety protection when working outdoors, which subjects the affected workers to the potentially life-threatening dangers of heat stress. To address this concern, employers should assess the demands of all jobs; complete a heat stress plan; post signage in areas where radiant heat is present; provide cool, shaded areas with water for breaks; and schedule strenuous jobs at cooler times of day. Workers should be provided with sun safety guidelines and personal protection like sunscreen, hats and recommendations for light, breathable clothing. For outdoor workers, regardless of the sector, appropriate heat exposure standards should be developed and implemented.

Manual material handling assessments should be completed to determine the risk factors that may lead to an OHS incident. Each risk factor is controlled by elimination or substitution, introducing administrative or engineering controls or providing equipment that mitigates the risk. Moreover, focused educational efforts should be undertaken to ensure knowledge and understanding of three basic employee rights: the right to know about actual and potential hazards they may encounter; the right to refuse unsafe work by bringing it to their supervisor’s attention and following the work refusal procedure; and the right to participate in proactively having a voice to address occupational health and safety issues in the workplace [36].

Although many of the respondents indicated they liked working as a farm worker, others noted they did not enjoy their role as a migrant farm worker, both verbally and in the questionnaire. With that in mind, employers should explore strategies to improve workplace morale in consultation with workers, including the potential establishment of a social committee to identify activities that could be implemented to create a more enjoyable workplace. Soccer matches, game nights, and sightseeing excursions are examples of events that would be well received as mentioned by some workers.

MAWs’ temporary status and dependence on their employer can create anxiety, which is exacerbated by loneliness and isolation in a new country far from family and friends. Employers should be prepared to provide access to mental health resources and training in multiple languages to address mental health and psychological safety concerns. Migrant workers should feel comfortable bringing issues to their supervisor without fear of reprisals such as being sent back home.

While many of these front-line OHS recommendations can be implemented by employers, advocates and scholars have argued that migrant workers’ underlying precarity is structured into the broader programs through which they are employed; as such, relying on employer compliance alone will be ineffective to ensure migrant workers feel empowered to access their rights [37,38,39,40,41]. Many migrant worker advocates as well as international organizations such as Amnesty International [42] and the United Nations [43] have noted the inherent vulnerability of these workers. Some improvements have been noted in a news release [44] both in Ontario workers’ compensation for injured workers in 2024 and federal retribution protection in 2022, as well as a federal toll-free hotline in 2021 where workers can reach out for assistance. The news release did go on to note that there is still room for further progress [44]. Calls for reform, such as status upon arrival or a pathway to permanent residency; open or flexible work permits; improved inspection and enforcement regimes; and enhanced funding for specialized services such as independent, accessible health care, such groups argue, are more fundamental shifts in policy needed to address the root causes of migrant worker vulnerability and to further safeguard their rights and protections.

## 6. Conclusions

Canada’s dependence on migrant agricultural workers is significant and only expected to continue increasing as an aging farm population and other complex factors point to persistent and growing chronic labor shortages in the agricultural sector. Despite the small sample size for this paper, this research adds to the evidence of numerous studies, which indicate the MAWs face structural and compounded vulnerabilities to OHS concerns, and lack sufficient access to protections and health care. Though the COVID-19 segment of the study is limited, the intention was to highlight that in addition to the various health, safety, and living conditions faced by migrant agricultural workers, COVID-19 is another health challenge that both MAWs and their respective employers must navigate. It is important to underscore that the study’s findings are exploratory and descriptive in nature, with the aim to provide deeper insights into the experiences of MAWs in Southern and Eastern Ontario, Canada. The findings reinforce and extend existing evidence on the topic rather than establishing significantly new definitive conclusions.

Collectively, this work supports the need to better understand and address the unique risk profile of this population to better serve their OHS needs. Indeed, the reliance on MAWs to ensure the food security of millions of people comes with a moral and legal obligation to ensure the health, safety and well-being of these workers.

## Figures and Tables

**Table 1:** Characteristics of respondents.

Variable	Subcategory	Count	Percentage (%)
Age	18 to 24	1	1.1
	25 to 34	29	31.5
	35 to 44	27	29.4
	45 to 54	25	27.2
	55 and above	9	9.8
Sex	Male	83	91.2
	Female	8	8.8
Education Level	Secondary	44	49.4
	Post-secondary	13	14.6
	Did not finish	32	35.9
Self-identify	Black	37	44.6
	Person of Color	18	21.7
	White	2	2.4
	Other	26	31.3
Country of Origin	Caribbean	38	40.9
	Mexico	54	58.1

**Table 2:** Number of health (e.g., noise or heat) and safety (e.g., falls or cuts) hazards experienced by the respondents.

Variable	Number of Incidents	Count	Percentage (%)
Health hazard experienced	0	59	71.1
	1 to 5	13	15.7
	6 to 10	10	12
	More than 10	1	1.2
Safety hazard experienced	0	67	72.8
	1 to 5	22	23.9
	6 to 10	3	3.3
	More than 10	0	0

**Table 3:** Working and living conditions with number and percentage responses. * For reporting purposes in Table 3, “I strongly agree” and “I agree” were combined and reported as Agree, while “I disagree” and “I strongly disagree” were combined and reported as disagree.

Variable	Subcategory	Count	Percentage (%)
My supervisors ask employees for their opinions before making decisions regarding health and safety	Agree	78	85.7
	Disagree	10	11.0
	Don’t Know	3	3.3
I have easy access to drinking water	Agree	84	92.3
	Disagree	7	7.7
	Don’t Know	0	0.0
I know how to work with chemicals that are sprayed on the crops	Agree	48	52.1
	Disagree	7	7.6
	Don’t Know	37	40.2
I am given protection to work in the rain or sun	Agree	76	85.45
	Disagree	11	12.4
	Don’t Know	2	2.2
I am not afraid of losing my job if I tell my supervisor I got hurt while working	Agree	82	89.1
	Disagree	8	8.7
	Don’t Know	2	2.2
I can take a paid sick day off work if I am sick	Agree	67	73.6
	Disagree	7	7.7
	Don’t Know	17	18.7

* Note: Cross-tabulation by language or education level did not provide any noteworthy difference.

## Data Availability

The research data is unavailable due to privacy restriction.
